# Die neue Gesetzgebung zur Organspende – Wirkung, Potenzial und Grenzen aus der Sicht klinisch tätiger Ärzt:innen

**DOI:** 10.1007/s00120-021-01645-y

**Published:** 2021-10-13

**Authors:** Zoë Fehring, Philip Boehme, Stefan Wirth, Leonard Fehring

**Affiliations:** 1 Fakultät für Gesundheit, Universität Witten/Herdecke, Alfred-Herrhausen-Straße 50, 58448 Witten, Deutschland; 2 Zentrum für Kinder- und Jugendmedizin, Universitätsklinikum Wuppertal, Universität Witten/Herdecke, Heusnerstraße 40, 42283 Wuppertal, Deutschland

**Keywords:** Transplantationsgesetz, Spendererkennung, Angehörigenbetreuung, Online-Register, Aufklärungsgespräch, Transplantation law, Donor recognition, Care of relatives, Online registry, Medical breefing

## Abstract

**Hintergrund:**

Die neuen Gesetze zur Organspende von 2019 und 2020 umfassen Regelungen zur Steigerung der Organspenderate. Dazu zählen Strukturverbesserungen, die Einführung eines Online-Registers zur Dokumentation der Entscheidung zur Organspende und die verbesserte Aufklärung der Bevölkerung. Zur Einschätzung der Maßnahmen durch unmittelbar betroffene klinisch tätige Ärzte ist bislang wenig bekannt.

**Methode:**

Es wurde eine zweistufige Sequential-mixed-method-Studie durchgeführt. Im Rahmen einer Online-Umfrage nahmen deutschlandweit 1235 Ärzte von über 90 Kliniken teil.

**Ergebnisse:**

Klinisch tätige Ärzte benennen größtenteils strukturelle Defizite als Ursachen für die niedrige Spenderrate in Deutschland. Weniger als die Hälfte der Ärzte, die mit Organspende in Kontakt kommen, findet sich ausreichend über Organspende ausgebildet. Ärztliche Aufklärungsgespräche haben wesentlichen Einfluss auf die Entscheidungsfindung von Patienten, werden aber kaum geführt. Da Patienten und Angehörige sich zu Lebzeiten zu wenig mit den Themen Tod und Organspende auseinandersetzten, führt die individuelle Entscheidungssituation oft zu Überforderung. Hausärzten kommt eine Schüsselrolle bei der Aufklärung zu. Von der Einführung eines Online-Registers erwarten nur wenige Befragte einen Einfluss auf die Organspenderate.

**Schlussfolgerung:**

Mit der neuesten Gesetzgebung wurde ein Großteil der von Ärzten benannten strukturellen Defizite aufgegriffen. Eine zusätzliche Steigerung der Organspenderate könnte durch professionalisierte Angehörigenbetreuung erreicht werden. Eine stärkere Thematisierung von Tod und Organspende in der Gesellschaft könnte im individuellen Fall die Entscheidungsfindung von Angehörigen erleichtern.

## Abstract (en)

**Background:**

The new German laws on organ donation of 2019 and 2020 intend to increase the organ donation rate. They include the optimization of organizational structures, the introduction of an online registry to document the patient’s will and measures to improve information of the public. To date, little is known about clinicians’ perspectives on these new laws.

**Methods:**

We conducted a two-stage sequential-mixed-method study. As part of an online survey, 1235 physicians from more than 90 hospitals throughout Germany participated.

**Results:**

Many clinicians name structural deficits as causes for the low donation rate in Germany. Less than half of the physicians who are exposed to organ donors regularly consider themselves to be adequately educated about organ donation. Physicians’ educational discussions with patients have a significant influence on patients’ decision-making but are rarely provided by doctors. In the decision-making situation, relatives are often overwhelmed because they have dealt with the topics of death and organ donation too little during their lifetime. General practitioners play a key role in educating and informing the population. Only a few respondents expect a positive impact on the organ donation rate by the introduction of an online donation registry.

**Conclusion:**

With the latest legislation, the most relevant structural deficits named by physicians have been addressed. An additional increase in organ donation rate could be achieved through professionalized care for relatives. A more frequent public discussion about death and organ donation could facilitate decision-making by relatives in individual cases.

Die neuen Gesetze zur Organspende von 2019 und 2020 sollen zur Steigerung der Organspenderate in Deutschland beitragen. Bisher ist wenig bekannt, wie unmittelbar betroffene klinisch tätige Ärzte die Änderungen der Gesetzgebung einschätzen. In diesem Artikel werden die Ergebnisse einer Befragung von mehr als 1000 klinisch tätigen Ärzten in Deutschland vorgestellt. Die Ergebnisse zeigen Grenzen der gesetzlichen Regelungen sowie weiteres Potenzial zur Steigerung der Organspendezahlen.

Im internationalen Vergleich der Organspenden pro 1 Mio. Einwohnern in 2020 lag Deutschland (11) weiterhin weit hinter Spitzenreitern wie Spanien (37,9) und den USA (38) [14]. Laut einer Repräsentativbefragung der Bundeszentrale für gesundheitliche Aufklärung (BZgA) von 2020 standen jedoch 82 % der Bevölkerung der Organspende positiv gegenüber und die Anzahl derer, die eine Entscheidung getroffen haben, ist von 56 in 2018 auf 62 % deutlich gestiegen [21, 22]. Die verhältnismäßig niedrige Zahl der Organentnahmen war Anlass für das Gesetz zur Verbesserung der Zusammenarbeit und der Strukturen bei der Organspende (GSZO) vom 22.03.2019, das Gesetz zur Stärkung der Entscheidungsbereitschaft bei der Organspende (OrgSpEG) vom 16.03.2020 und den Gesetzesentwurf zur Einführung der doppelten Widerspruchslösung, den der Bundestag im Januar 2020 ablehnte.

Über die Gesetzesänderungen wurde in ärztlichen Fachzeitschriften umfänglich berichtet [4, 5, 16, 24]. Der Gesetzgeber reagiert damit auf strukturelle Probleme in Krankenhäusern und versucht, die Aufklärung der Bevölkerung zu verstärken und den Spenderwillen besser zu dokumentieren.

Klinisch tätigen Ärzten kommt bei der Verwirklichung von Organspenden eine Schlüsselrolle zu. Ihnen obliegt die Umsetzung der neuen Gesetze in den Kliniken, die Entscheidung im Einzelfall und die Kommunikation mit Angehörigen. Sie können die Wirkung der getroffenen Maßnahmen erfahrungsbasiert abschätzen. Bisher wurde die ärztliche Perspektive auf die Gesetzesänderungen aber nur wenig erforscht. Diese nach unserer Kenntnis größte wissenschaftliche Untersuchung in Deutschland geht der Frage nach, ob die aus ärztlicher Sicht relevanten Problemfelder von den gesetzlichen Neuerungen abgedeckt werden und ob weitergehendes Potenzial zur Steigerung der Organentnahmezahlen besteht.

## Methode

Die vorliegend dargestellte Studie im Sequential-mixed-method*-*Design umfasste eine qualitative und quantitative Phase [6]. Für die qualitative Phase wurden auf der Basis wissenschaftlicher Vorrecherchen Leitfragen zur strukturierten Durchführung persönlicher Interviews mit vorwiegend offenen Fragen entwickelt. Anhand derer wurden klinisch tätige Ärzte mit gezielten Stichproben interviewt (Chirurgen, Anästhesisten, Intensiv- und Notfallmediziner und Transplantationsbeauftragte). Dabei wurden die persönliche Einstellung zur Organspende sowie Erfahrungen mit den Strukturen und finanzielle Aspekte berücksichtigt. Nach Transkription der aufgezeichneten Interviews [8] wurde eine qualitative Inhaltsauswertung in einem explikativen Ansatz nach Mayring durchgeführt [19]. Mit den Ergebnissen aus der qualitativen Phase wurde für die quantitative Phase in LimeSurvey© Version 3.25.3 + 201.208 (LimeSurvey GmbH, Hamburg) ein Online-Fragebogen mit 32 geschlossenen Fragen (Beantwortung überwiegend mit 5‑stufiger Likert-Skala) entwickelt. Dabei wurde die Einstellung zu Aufklärung, Krankenhausstrukturen, Finanzierung und Verbesserungsvorschläge thematisiert. Die Umfrage wurde vorab auf Verständlichkeit, Konsistenz und Befragungsdauer an 5 Probanden aus unterschiedlichen Krankenhäusern überprüft [23]. Der Link zur Online-Umfrage wurde an alle Ärzte der Helios-Kliniken Deutschland (11.000 Empfänger) versendet. Antworten wurden zwischen Oktober 2020 und März 2021 erhoben und einer Plausibilitätsprüfung unterzogen. 46 % der Helioskliniken in Deutschland hatten 2020 organspendebezogene Kontakte; insgesamt wurden an den Kliniken 47 Organspender verwirklicht [10]. Die deskriptive statistische Auswertung erfolgte mit Microsoft Excel (Version 2002) (Microsoft Corp., Redmond, WA, USA); bei den Likert-Fragen wurden Antworten > 3 als Zustimmung gewertet und der Zustimmungsanteil berechnet. Für Ärzte, die laut eigener Aussage regelmäßig mit potenziellen Organspendern in Kontakt kommen, und Chefärzte erfolgte eine Subgruppenauswertung.

## Ergebnisse

In Phase 1 wurden bis zur inhaltlichen Sättigung 17 Ärzte aus 8 Krankenhäusern interviewt. Die Befragten thematisierten übergreifend vier Problemfelder: mangelhafte Aufklärung der Bevölkerung, Unzugänglichkeit von Angehörigen in der Entscheidungssituation, eine fehlende Widerspruchslösung und eine unzureichende Vergütung der Organentnahme für Kliniken. Darüber hinaus problematisierten alle Befragten, dass die Angehörigengespräche sensible Kommunikation und ausreichend Zeit erfordern und deshalb im eng getakteten Arbeitsalltag nur unzureichend geführt werden können.

An der Online-Umfrage in Phase 2 nahmen 1235 Ärzte teil (Antwortquote 11 %). Davon konnten 1049 Datensätze nach Plausibilitätsprüfung verwendet werden. 971 Teilnehmer füllten den Fragebogen vollständig aus, konnten jedoch Fragen durch die Auswahl von „Weiß nicht“ auslassen. An der Umfrage nahmen Ärzte aus über 90 Kliniken teil. 49 % der Befragten gaben an, regelmäßig Kontakt zu potenziellen Organspendern zu haben (Tab. 1).

**Table 1:** 

Kategorie	Dimension	
Stichprobengröße	Teilnehmer in der Wertung	1049
Art des Krankenhauses	Krankenhaus der Grundversorgung	18 %
	Krankenhaus der Regelversorgung	43 %
	Krankenhaus der Maximalversorgung	39 %
Häufigkeit von Transplantationen in den befragten Kliniken (Selbstauskunft)	Weniger als einmal im Jahr	38 %
	Jährlich	15 %
	Halbjährlich	36 %
	Monatlich	9 %
	Mehr als einmal im Monat	3 %
Position	Assistenzarzt	29 %
	Oberarzt	26 %
	Facharzt	18 %
	Leitender Oberarzt	11 %
	Chefarzt	15 %
Erfahrung	Mittlere Dienstzeit	16,5 Jahre
	Standardabweichung mittlere Dienstzeit	10,8 Jahre
	Anteil der Teilnehmer, die in ihrer Tätigkeit potenziell mit Organspendern in Kontakt kommen	49 %
Verteilung der Facharztgruppen	Allgemeinmedizin	1 %
	Anästhesiologie	21 %
	Chirurgie	20 %
	Gynäkologie	4 %
	Innere Medizin	19 %
	Neurologie	5 %
	Pädiatrie	7 %
	Andere	23 %

Die Einflussfaktoren auf die Organspenderate lassen sich in zwei Kategorien klassifizieren: Die Identifizierung potenzieller Spender und Realisierung einer Organentnahme sowie die allgemeine Spendebereitschaft der Bevölkerung.

### Identifizierung und Realisierung potenzieller Organspender

#### Die meisten Ärzte sind offen für Organspende

**Figure 1:**
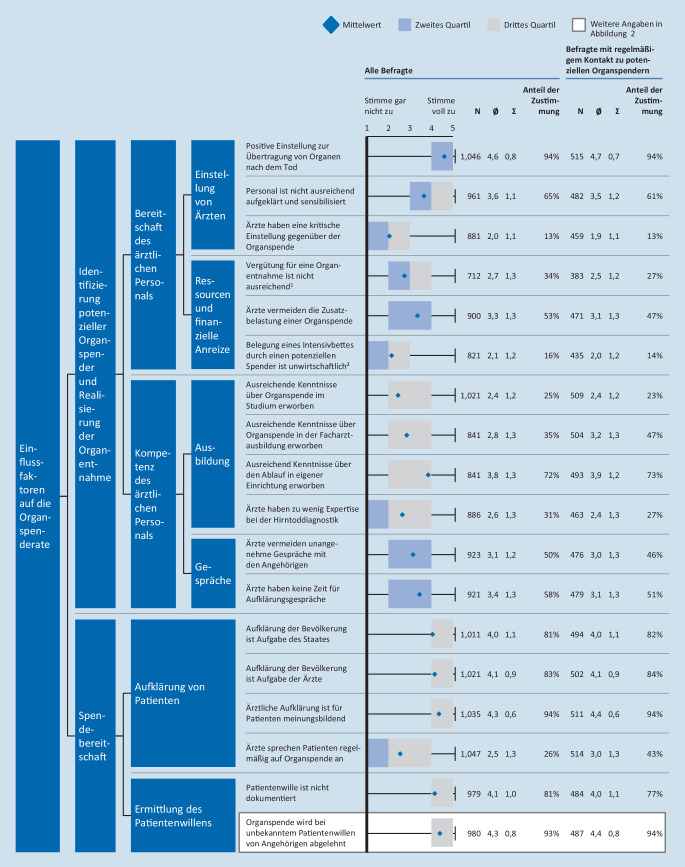

Insgesamt geben 94 % der Befragten an, der Organspende grundsätzlich positiv gegenüberzustehen. Von den Befragten mit regelmäßigem Kontakt zu potenziellen Organspendern geben 26 % an, dass nicht alle potenziellen Organspender in ihrer Klinik identifiziert werden. Als wichtiger Grund für eine Nicht-Realisierung von potenziellen Organspendern wird eine fehlende Sensibilisierung des Personals genannt (65 % Zustimmung). Die Ablehnung der Organspende durch medizinisches Personal hat für die Meldung potenzieller Spender jedoch kaum Bedeutung (13 % Zustimmung; Abb. 1).

#### Die Vergütung ist angemessen

Nur 27 % des Befragten mit regelmäßigem Kontakt zu potenziellen Organspendern sehen eine Ursache für die nicht erfolgte Meldung in der mangelnden Vergütung. Die unwirtschaftliche Belegung von Intensivbetten durch Organspender spielt in derselben Gruppe nur bei wenigen eine Rolle (14 % Zustimmung); unter Chefärzten nur bei 9 % (Abb. 1).

#### Viele Ärzte finden sich nicht ausreichend ausgebildet

Nur 25 % aller Befragten geben an, dass sie in ihrem Studium ausreichend über Organspende informiert wurden, 35 % durch die Facharztausbildung. Bei Befragten mit regelmäßigem Kontakt zu potenziellen Organspendern liegt der Anteil der durch die Facharztausbildung ausreichend Informierten bei 47 %. 27 % der Befragten mit regelmäßigem Kontakt zu potenziellen Organspendern sind sich bei den Abläufen einer Organspende in ihrer eigenen Klinik unsicher (Abb. 1). Nur 50 % wussten, wer der Transplantationsbeauftragte ihrer Klinik ist.

#### Eine angemessene Gesprächsführung im Klinikalltag stellt eine Herausforderung dar

Dass potenzielle Spender nicht gemeldet wurden, liegt laut 58 % unter anderem daran, dass zu wenig Zeit für Aufklärungsgespräche zur Verfügung steht. 47 % der Befragten mit regelmäßigem Kontakt zu potenziellen Organspendern geben an, dass mögliche Spender wegen der Zusatzbelastung für das Personal nicht gemeldet werden. 46 %, dass unangenehme Gespräche mit Angehörigen gemieden werden (Abb. 1).

### Spendebereitschaft

#### Das Aufklärungsgespräch ist wichtig, wird aber nur selten geführt

Laut 94 % der Befragten ist das Aufklärungsgespräch meinungsbildend für Patienten. Nur 26 % sprechen das Thema Organspende jedoch regelmäßig bei ihren Patienten an. Unter den Befragten mit regelmäßigem Kontakt zu potenziellen Organspendern sind es 43 % (Abb. 1). 60 % der Befragten sehen die Zuständigkeit für die Aufklärung von Patienten bei Hausärzten. Andere sehen Ärzte der Intensivstation (16 %), Oberärzte im Krankenhaus (11 %), Stationsärzte (3 %) und niedergelassene Fachärzte (1 %) in der Hauptverantwortung.

#### Die Ermittlung des Patientenwillens ist aufwendig

**Figure 2:**
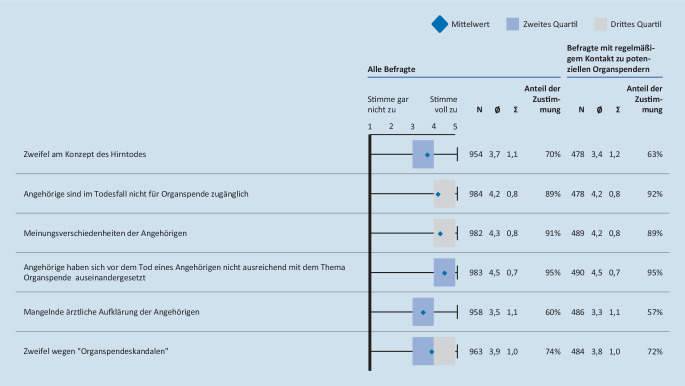

Dass eine Organentnahme bei einem potenziellen Spender nicht realisiert wurde, liegt laut 77 % der Befragten mit regelmäßigem Kontakt zu potenziellen Organspendern auch daran, dass die Entscheidung des Patienten über die Organ- und Gewebespende nicht dokumentiert ist. Gibt es keinen schriftlich fixierten Willen des Verstorbenen, wird die Organentnahme häufig von Angehörigen abgelehnt (94 % Zustimmung; Abb. 1). Unter den Gründen für die Ablehnung durch Angehörige wird von den Befragten mit regelmäßigem Kontakt zu potenziellen Organspendern die fehlende Auseinandersetzung mit Organspende angegeben (95 % Zustimmung). Etwa 90 % sehen Gründe in der fehlenden Zugänglichkeit und in Meinungsverschiedenheiten der Angehörigen (Abb. 2).

### Maßnahmen zur Verbesserung der Identifikation potenzieller Spender

#### Organisatorische Entlastung der Ärzte könnte Organspenderate verbessern

**Figure 3:**
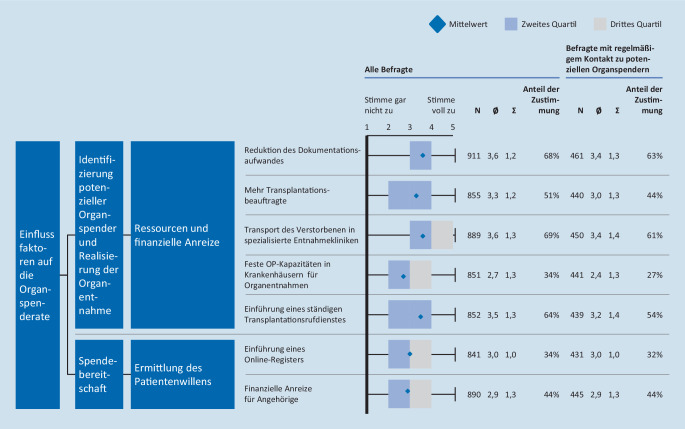

Es gingen 69 % aller Befragten davon aus, dass der Transport von Verstorbenen in spezialisierte Organentnahmekliniken die Organspenderate verbessern könnte. 64 % Zustimmung erhielt zudem die Einführung einen ständigen Transplantationsdienstes, der bei Bedarf die Explantation durchführen kann. 51 % der Befragten hielten mehr Transplantationsbeauftragte für erforderlich. Weniger Zustimmung fanden die Einplanung fester OP-Kapazitäten für Organentnahmen (34 %) und die Einführung eines Online-Registers zur Erfassung der Entscheidung zur Organ- und Gewebespende (34 %). Finanzielle Anreize für Angehörige etwa durch Erstattung von Bestattungskosten sind laut 44 % der Befragten geeignet, die Organspendezahlen zu steigern, aber nur für 24 % ethisch vertretbar (Abb. 3).

## Diskussion

### Strukturprobleme sind überwiegend behoben

Die geringen Organspendezahlen werden in Deutschland schon länger auf Strukturprobleme zurückgeführt, wie das Erkennungs- und Meldedefizit in Krankenhäusern, eine fehlende Expertise bei der Hirntoddiagnostik und den Bedarf an Schulung und Ausbildung des Personals [3, 26]. Mit dem GSZO reagierte der Gesetzgeber auf diese bekannten Strukturprobleme u. a. durch die Implementierung und Freistellung von Transplantationsbeauftragten und die Einführung eines neurologischen Konsiliardienstes. Das Hinzuziehen von intensiv geschulten Transplantationsmedizinern und die Zentralisierung der Organspende auf wenige Kliniken führte bereits in Spanien zum Erfolg [18, 28]. Aus ärztlicher Sicht gibt es in Deutschland aber nach wie vor zu wenig Transplantationsbeauftragte, die zudem nicht im klinischen Alltag präsent und eingebunden sind. Darüber hinaus stellen viele Kliniken Transplantationsbeauftrage weiterhin nicht von ihren übrigen Aufgaben frei [25]. Eine Zentralisierung etwa durch die Einführung spezialisierter Entnahmekliniken wäre aus ärztlicher Sicht erfolgversprechend. Der tatsächliche Einfluss bleibt aber fraglich, da nur 27 % der Befragten angeben, dass feste OP-Kapazitäten zur Steigerung der Organspenderate beitragen. Durch eine Zentralisierung könnte zudem das Wissen über Organspende in peripheren Kliniken verloren gehen und die Sensibilisierung des Personals abnehmen. Über den Einfluss des Transports des Verstorbenen auf die Entscheidung der Angehörigen ist wenig bekannt. Die Erhöhung der Vergütung einer Organentnahme durch das GSZO ist aus ärztlicher Sicht angemessen, dennoch konnte hierdurch kein relevanter Anstieg der Organspendezahlen festgestellt werden.

### Verbesserung der ärztlichen Ausbildung zu begrüßen

Durch das OrgSpEG wurde die Organspende stärker in der Approbationsordnung für Ärzte verankert. Dass verstärkte Ausbildung von medizinischem Personal im Bereich Organspende erforderlich ist, zeigt der erhebliche Mangel an Kenntnissen bei Ärzten mit regelmäßigem Kontakt zu Organspende. Allerdings werden sich die Folgen erst in einigen Jahren zeigen, wenn Ärzte nach der neuen ÄApprO ausgebildet wurden. Die Stärkung der Rolle des Transplantationsbeauftragten in Kliniken ist daher sinnvoll. Diese können Fachwissen vermitteln und klinisches Personal sensibilisieren. Der geringen Kompetenz im Umgang mit Organspende könnte durch Fortbildungen und die Integration der Organspende in die Facharztausbildung begegnet werden.

### Intensive Betreuung von Angehörigen durch Transplantationsbeauftrage ist wünschenswert

Mit dem GSZO wurde den Transplantationsbeauftragten die Verantwortung für eine „angemessene“ (§ 9b Abs. 2 Nr. 2 TPG) Angehörigenbegleitung vor und während der Organentnahme übertragen. Deffner et al. [7] kritisierten, dass diese Regelung nicht detailliert genug ist und keine Betreuungskonzepte vorgesehen sind. Die Zustimmung der Angehörigen hängt aber entscheidend von einem professionellen, respektvollen und angemessenen ärztlichen Gespräch über eine mögliche Organspende ab. Das setzt insbesondere voraus, dass die Gesprächspartner nicht wechseln und ein vertrauensvolles Verhältnis besteht [1, 13, 15, 17, 20]. Schon vor dem GSZO stellte die Deutsche Stiftung Organtransplantation (DSO) Angehörigenbegleitung und Fortbildungsangebote zur Verfügung: Im Idealfall sollen Angehörigengespräche von geschulten Ärzten und dem Koordinator geführt werden [9]. In dieser Studie konnte gezeigt werden, dass Ärzten der Einfluss von Gesprächen auf die Entscheidung von Angehörigen bewusst ist. Häufig besteht jedoch zu wenig Raum, Gespräche mit Angehörigen mit der benötigten Ruhe und Zeit im Klinikalltag angemessen unterzubringen. Ein nicht unerheblicher Teil der Ärzteschaft empfindet die Gespräche zudem als unangenehm. Auf den erheblichen Einfluss von Angehörigengesprächen auf die Organspenderate wurde in anderen Ländern bereits umfänglich reagiert. In Großbritannien stehen etwa spezialisierte Krankenschwestern rund um die Uhr für die Betreuung von Angehörigen bereit. Sie sind speziell für Organspende geschult, führen Gespräche mit Angehörigen und helfen bei der Entscheidungsfindung [13]. In den USA wurden bereits 2015 Kommunikationstrainings vorgestellt („communicating effectively about donation“ [CEaD]), mit Hilfe derer die Zustimmungsraten der Angehörigen zur Organspende und die Gesamtqualität der Erfahrung mit Organspende erheblich verbessert werden konnte [27]. Insofern bedarf es auch in Deutschland vertiefter und verbindlicher Konzepte und Regelungen zur Angehörigenbetreuung mit Kommunikationstrainings und Schulungen für die Betreuung von Angehörigen im Fall einer Organspende.

### Ein Online-Register kann eine fehlende Patientenentscheidung nicht überwinden

Mit der Ablehnung der Widerspruchslösung Anfang 2019 bleibt der nicht dokumentierte Wille ein entscheidendes Hindernis zu mehr Organspenden in Deutschland. Die Überforderung, fehlende Zugänglichkeit und Auseinandersetzung mit dem Thema sowie Meinungsverschiedenheiten von Angehörigen gestalten die Angehörigengespräche schwierig und sind nicht zu vernachlässigende Gründe für die Nicht-Realisierung einer Organentnahme. Mit der Einführung eines Online-Registers zur Dokumentation der Entscheidung zur Organ- und Gewebespende versucht das OrgSpEG hier Abhilfe zu schaffen. Nur jeder Dritte Arzt erhofft sich jedoch von der Einführung einen merklichen Effekt. Trifft der Patient zu Lebzeiten keine Entscheidung, hilft auch ein Online-Register nicht weiter und die Angehörigen müssen eine Entscheidung treffen. Vielmehr sind Aufklärung und Etablierung einer „Kultur der Organspende“ [5] in der Bevölkerung entscheidend: beim Spitzenreiter Spanien gehört Organspende selbstverständlich zu den Entscheidungen am Lebensende [28]. Eine dauerhafte öffentliche Debatte wäre daher auch in Deutschland zu begrüßen.

### Sind die Hausärzte der Schlüssel zum Erfolg?

Auf die Notwendigkeit einer breiteren Aufklärung der Bevölkerung wurde schon oft hingewiesen [11], gleichzeitig konnte jedoch auch gezeigt werden, dass Kampagnen in der Öffentlichkeit wenig Erfolg haben [12]. Das OpgSpEG versucht daher die Aufklärung der Bevölkerung stärker in den Fokus zu rücken, etwa durch Ausgabe von Aufklärungsmaterial durch Meldebehörden und beim Erwerb der Fahrerlaubnis. Weiterhin sollen Hausärzte künftig bei Bedarf ihre Patienten alle 2 Jahre über die Organ- und Gewebespende ergebnisoffen beraten. Aus ärztlicher Sicht knüpft dies ebenfalls an die fehlende Auseinandersetzung mit der Organ- und Gewebespende an. Die stärkere Einbindung von Hausärzten in die Aufklärung der Bevölkerung ist daher grundsätzlich zu begrüßen. Ob Hausärzte die Auffassung teilen, ist bisher nicht bekannt. Eine landesweite Umfrage unter Hausärzten hat gezeigt, dass > 80 % der Hausärzte Informationsmaterial auslegen, jedoch nur knapp 40 % ihre Patienten auf Organspende aktiv ansprechen [2]. Ob der Besuch beim Hausarzt die Auseinandersetzung mit Organspende zulässt, Hausärzte ausreichend informiert sind und welche weiteren Voraussetzungen nötig sind, sollte vorausschauend geprüft werden, damit der erhoffte Einfluss auf die Organspendezahlen gelingen wird.

## Ausblick

Die gesetzlichen Neuregelungen zur Erhöhung der Organspenden in Deutschland bringen grundsätzlich jene Verbesserungen, die klinisch tätige Ärzte im Blick auf strukturelle Defizite benennen, wie die Freistellung von Transplantationsbeauftragten, die verstärkte Integration der Organspende in die ärztliche Ausbildung und breitere Aufklärung der Bevölkerung durch Beratungsangebote der Hausärzte. Die Einführung eines Online-Registers zur Dokumentation der Entscheidung zur Organspende wird aus ärztlicher Sicht hingegen nicht den gewünschten Effekt haben. Entscheidender ist eine bessere Aufklärung der Bevölkerung und professionalisierte Betreuung von Angehörigen, die sensible und angemessene Gesprächsführung gestattet. Unter welchen Bedingungen Hausärzte über Organspende aufklären können und ob sie den erforderlichen Aufklärungserfolg erreichen werden, bleibt offen.

## Fazit für die Praxis

- Mit den gesetzlichen Neuregelungen zur Erhöhung der Organspenden in Deutschland wurden die von klinisch tätigen Ärzten benannten strukturellen Defizite aufgegriffen.
- Transplantationsbeauftragte können wichtige Aufgaben bei der Schulung und Sensibilisierung des ärztlichen Personals übernehmen.
- Die Zentralisierung der Organentnahme über spezialisierte Teams oder Kliniken ist darüber hinaus geeignet, die Ärzteschaft zu entlasten und die klinischen Prozesse zu vereinfachen.
- Bei der Etablierung der Themen Tod und Organspende in der Gesellschaft kommt der Ärzteschaft eine Schlüsselrolle zu.
- In professionalisierter Angehörigenbetreuung liegt Potenzial zur Steigerung der Organspenderate.
